# Characterization of airborne bacterial diversity in conventional hen houses, enriched colonies and aviaries, and link between possible bioaerosol sources

**DOI:** 10.1016/j.psj.2025.105217

**Published:** 2025-04-29

**Authors:** M.-W. St-Germain, M. Veillette, V. Létourneau, A.D. Larios Martínez, S. Godbout, M. Boulianne, C. Duchaine

**Affiliations:** a Département de biochimie, de microbiologie et de bio-informatique, Faculté des sciences et de génie, Université Laval, Québec, Canada; b Centre de recherche de l’Institut universitaire de cardiologie et de pneumologie de Québec, Québec, Canada; c Research and Development Institute for the Agri-Environment (IRDA), Québec, Canada; d Department of Clinical Sciences, Faculty of Veterinary Medicine, Université de Montréal, Saint-Hyacinthe, Canada; e Chaire en recherche avicole de l'Université de Montréal, Faculty of Veterinary Medicine, Université de Montréal, Saint-Hyacinthe, Canada; f Canada Research Chair on Bioaerosols, Québec, Canada

**Keywords:** laying hens, alternative hen houses, 16 s rRNA gene, bacterial diversity, bioaerosols, feces

## Abstract

**Background:**

Canada’s transition toward alternative housing systems for laying hens may have an impact on bioaerosol content and concentrations in those environments. This project aimed to characterize the airborne bacterial diversity in six conventional hen houses, six enriched colonies and six aviaries. The bacterial diversity found in bioaerosols was also compared to the diversity found in feces or litter samples from each corresponding housing type to investigate similarities between possible bioaerosol sources and bioaerosols.

**Results:**

Specific richness (S_obs_) and CHAO1 indexes were higher in air samples from conventional hen houses and enriched colonies, compared to their corresponding fecal or litter samples, which was not the case for aviaries samples. No significant differences were found between the Shannon and inverse Simpson (InvSimpson) indexes of air samples, compared to their corresponding fecal or litter samples. *Firmicutes* were the dominant phyla in all samples, followed by *Actinobacteria*. Dominant genera were *Lactobacillus,* unclassified *Lanchnospiraceae,* unclassified *Actinomycetales*, unclassified *Clostridales* and unclassified *Ruminococcaceae*. OTUs (Operational Taxonomic Units) were associated with hen microbiota and gut microbiota, and soil. Homogeneity of molecular variance analyses (HOMOVA) revealed significant differences between air samples from aviaries, compared to air samples from conventional and enriched cage houses. Significant differences were found between air and fecal or litter samples from conventional hen houses and enriched colonies, but not among aviary samples.

**Conclusions:**

Findings highlight the effects of housing types on airborne bacterial diversity, and similarities in bacterial diversity between air and fecal or litter samples from three types of husbandry. Most dominant OTUs were shared across all samples, but were different in proportions, which may account for the differences in alpha and beta diversities. The overlap in bacterial diversities between air and litter samples collected in aviaries brings out the contribution of litter to ambient bioaerosols.

## Introduction

To comply with new animal welfare practices, Canada’s egg producers have begun a phase-out of conventional hen houses and a transition toward alternative housing systems (National Farm Animal Care Council, and Egg Farmers of Canada, 2017). Laying hens in conventional husbandry are housed in small groups on wire floor cages, with *ad libitum* access to water and feeding according to body weight. Alternative houses provide larger space for each hen and enrichment to facilitate natural behaviors such as wing stretching, nest-box egg laying, perching and scratching (Appleby and Hughes, 1991; Hartcher and Jones, 2017; Hester, 2005). Canada’s Code of Practice for the Care and Handling of Pullets and Laying Hens includes two main types of alternative hen houses: enriched colonies and cage-free houses (single-tier or multi-tier) (National Farm Animal Care Council, and Egg Farmers of Canada, 2017). Enriched colonies share many characteristics with conventional hen houses as animals are housed in cages in small groups, with access to food and water. Enriched colonies also include access to perches, scratching and nesting areas. Cage-free houses are hen houses providing food, water and nest boxes on one or multiple perch levels, and a litter floor made of sand, straw or woodchips.

Cage-free housing systems for laying hens have been associated with increased ambient dust and bioaerosol concentrations when compared to conventional housing systems (Appleby and Hughes, 1991; Arteaga et al., 2015; Le Bouquin et al., 2013). Bioaerosols in poultry facilities originate from the animals (skin flakes, feathers, down), animal feces, feed and bedding material in the presence of a litter floor (Cambra-López et al., 2010; Ellen et al., 2000; Just et al., 2009). Occupational exposure to bioaerosols in conventional laying hen houses and broiler operations has already been linked to the development of non-specific respiratory symptoms in workers (Donham et al., 2000; Just et al., 2009; Kirychuk et al., 2003; Senthilselvan et al., 2011). However, health risks associated with the presence in bioaerosols of potentially pathogenic bacteria for workers or flocks remain poorly understood.

The bacterial content in alternative hen house air also remain largely uncharacterized and few studies have previously examined the bacterial diversity conventional laying hen houses using culture-independent methods such as the amplification through sequencing of variable regions of the 16S rRNA gene (Cui et al., 2023; Just et al., 2011; Yan et al., 2023). The 16S rRNA gene amplicon-based sequencing allows the analysis of a high number of sequences within environmental samples and the bypassing of culture-dependent methods’ bias and limitations(Delort, 2017). High-throughput sequencing has thus revealed the presence of the phyla *Firmicutes, Bacteroidetes, Proteobacteria, Cyanobacteria, Fusobacteria* and *Actinobacteria*, and genera *Lactobacillus,* unknown *Bacteroidetes, Turicibacter, Facklamaia, Corynebacterium, Fusobacterium, Aerococcus, Comamonas, Faecalibacterium, Enterococcus, Olsenella, Joetgalicoccus* and *Steriotrophomonas* in conventional laying hen facilities (Cui et al., 2023).

As airborne bacterial diversity was not yet characterized in alternative hen house (enriched colonies and aviaries) in Eastern Canada, this project aimed to analyze the bacterial diversity found in air samples from those environments compared to conventional laying hen house, using high flow air sampling and high-throughput amplicon sequencing of the 16S rRNA gene. The bacterial content of fecal and litter samples was also analyzed to correlate bioaerosols with their potential sources. Housing type was expected to have an impact on airborne bacterial diversity since alternative hen houses provide freedom of movement, and aviaries a litter floor, which could represent unique sources of bioaerosols due to the presence of feces, feed, and litter substrate. Overlaps between bacterial communities found in bioaerosols and their corresponding source were expected in all hen houses. The findings in this study may provide insight on the impact of alternative hen houses on bioaerosol composition, in the context of transition toward alternative housing systmes in the Canadian egg industry. They may guide future studies aiming to monitor specific bacteria that are relevant for human, animal or environmental health.

## Methods

### Ethics statement

As the protocols did not involve the manipulation of hens or alteration of their living environment, this project did not require the approval of the local committee for animal protection (CPA) at Université Laval. Written authorization was obtained from the manager of operations in each hen house to access the building and conduct air and feces/litter samplings. Authorization forms were signed by both managers and the head researcher of the present study.

### Laying hen houses

Bioaerosols, feces and litter samples were taken in six conventional hen houses, six enriched colonies and six aviaries (multi-tier cage-free hen houses) located within a radius of 200 km from Quebec City. All buildings were mechanically ventilated and housed hens of approximately 50 weeks of age and older. Conventional hen houses had a median volume of 2,741 m³ (min: 1,425 m³; max: 3,976 m³) and housed a median number of 25,025 hens (min:16,510 hens; max: 44,028 hens), while enriched colonies had a median volume of 4,139 m³ (min: 1,752 m³; max: 12,488 m³) and housed a median number of 18,499 hens (min: 13,919 hens; max: 92,000 hens). Aviaries’ median volume was 4,500 m³ (min: 2,743 m³; max: 7,604 m³), and a median number of 18,750 hens per aviary (13,693 hens; max: 22,978 hens). Other building characteristics and environmental conditions are detailed in St-Germain et al. (2024b). Each hen house was visited once during the cold season (October to March, inclusively) to ensure the buildings would operate at minimal ventilation rates.

### Bioaerosol, Feces and Litter Sampling

Bioaerosols were sampled on electret filters using the SASS®3100 Dry Air Sampler (300 L/min, Research International, Monroe, Washington, USA). Three samples (3 × 10 min, 3 m^3^) were taken at moments spread over four hours after 10 a.m., in a single location inside each building and at approximately 1.1 m above the ground. Field blanks were taken during each sampling visit, and consisted in exposing a filter, for a few seconds, to air inside the hen houses without active sampling. Filters were stored at 4°C until the extraction of air particulates.

Two 25 g fecal samples (or litter samples, from aviaries) were taken at random locations within the same hen house row as the air samplers in each hen house using sterile plastic scoops and sterile plastic bags. Feces from the conveyer belts were sampled a day to at most four days after the activation of manure belts in conventional hen houses, and after at most 7 days after manure removal in enriched colonies, as reported by barn managers. A field blank in each building was also conducted by exposing a scoop and a bag to the air of the visited hen house. Fecal and litter samples were stored at 4°C until processing.

### Sample processing and DNA Extraction

Two days following a sampling campaign, air samples were extracted from the electret filter using the SASS®3010 Particle Extractor (Research International, Monroe, Washington, USA) and 7 mL of a sterile phosphate buffer (138 mM sodium chloride, 10 mM sodium phosphate, 2.5 mM potassium chloride, 0.5 % Triton X-100, < 0.1 % sodium azide). Aliquots (3 × 1 mL) of each air sample were then filtered on 0.2 µm porosity polycarbonate membranes (GTBP02500, Millipore, Merck KGaA, Darmstadt, Germany) using vacuum filtration units, as previously described by Mbareche and collaborators (2019). Each filter was placed into individual 1.7 mL sterile polypropylene tubes and kept at −20°C until DNA extraction.

Fecal and litter samples were also treated within two days following the sampling visit. For each sample, 25 g of feces or litter were mixed for 1 minute with 200 mL of phosphate-buffered saline solution 1X (PBS 1X) + 0.05 % Tween®20 using the AES Laboratoire’s paddle blender (bioMérieux, series 90410857, 50/60 Hz, Marcy-l’Étoile, Lyon, France) for samples from barns 1 to 12 inclusively, and the Fisherband™ Triplemix Paddle Blender (230 rpm, fisher scientific, Hampton, New Hampshire, USA) for samples from barns 13 to 18 inclusively. The scoop and bag of each field blank were rinsed with the same amount of PBS 1X +0.05 % Tween®20 and homogenized. Aliquots (3 × 1 mL) of each sample were centrifuged at 14,000 x g for 10 minutes at room temperature. Pellets were kept at – 20°C until DNA extraction.

DNA extraction was performed using the DNeasy® Powerlyser® Powersoil Kit (QIAGEN, Germantown, Maryland, USA). Additional steps were added to the manufacturer’s protocol to extract DNA from the filtered air samples (Mbareche et al., 2019b). 750 µL of Powerbead Solution (QIAGEN) and a 3 mm sterile tungsten bead were added to each tube containing a filtered air sample. Samples were then shaken at 30 beats per second for 20 min in a bead-beating machine (Mixer Mill MM301, Retsch, Düsseldorf, Germany), in order to pulverize the filters. Pulverized filters were then used for DNA extraction, following the manufacturer’s recommendation. DNA extracts of air, feces, and litter samples were kept at −20°C until further analysis.

### Library Preparation and High-Throughput Sequencing

Prior to high-throughput sequencing, equimolar pooling of triplicate air samples and duplicate fecal and litter samples from each hen house was performed in 96-well plates for a final volume of 30 µL per well (3 × 10 µL for air samples, and 2 × 15 µL for fecal and litter samples). Field blanks were pooled according to their hen house type of origin (conventional hen houses, enriched colonies and aviaries), for a final volume of 30 µL per well (6 × 5 µL). The 96-well plates were then sent to the Plateforme d’analyse génomique (IBIS, Université Laval, Québec, QC, Canada) for the amplification of specific variable regions of the 16S rRNA gene and high-throughput sequencing.

Library preparation was conducted using a two-step dual-indexed PCR designed for the Illumina® instruments(San Diego, California, USA) was used to amplify the V6-V8 regions of the 16 s rRNA gene, as cited in Comeau and collaborators (Comeau et al., 2011) (Table 1). The first PCR reaction was carried out in a volume of 25 µL containing 1x Q5 buffer (NEB, Ipswish, Massachusetts, USA), 0.25 µM of forward and reverse primer, 200 µM of each dNTP, 1 U of Q5 High-Fidelity DNA polymerase (NEB) and 1 µ L of DNA template. PCR thermoprotocol was conducted as follows: initial denaturation at 98°C for 30 s, 35 cycles of denaturation at 98°C for 10 s, annealing at 55°C for 10 s, extension at 72°C for 30 s and a final extension at 72°C for 2 minutes. Quality control of the first amplicons was performed on a 1 % agarose gel. The purified products were then diluted 50- to 100-fold and used as templates for the second dual-indexed PCR. A twelve-cycle thermoprotocol identical to the first was conducted, samples were purified on a 1 % agarose gel, and their quality was validated using the DNA7500 Bioanalyzer chip (Agilent®, Santa Clara, California, USA). Amplicons were then quantified using the Nanodrop® 1000 (Thermo Fisher Scientific, Waltham, Massachusetts, USA) before being sequenced using the Illumina® MiSeq machine.

**Table 1:** PCR primers used for the amplification of the 16S rRNA gene.

Primer sets	Sequence from 5′ to 3′
1st PCR primers	F:5′-ACACTCTTTCCCTACACGACGCTCTTCCGATCTACGCGHNRACCTTACC-3′
	R: 5′-GTGACTGGAGTTCAGACGTGTGCTCTTCCGATCTACGGGCRGTGWGTRCA-3′
2nd PCR primers	F: 5′-AATGATACGGCGACCACCGATCTACA[index1]ACACTCTTTCCCTACACGAC-3′
	R: 5′-CAAGCAGAAGACGGCATACGAGAT[index2]GTGACTGGAGTTCAGACGTGT-3′

^a^Primers used in this study contain sequences specific to Illumina® and are protected by intellectual property. (Oligonucleotide sequences © 2007–2023 Illumina, Inc. All rights reserved. Derivative works created by Illumina customers are authorized for use with Illumina instruments and products only. All other uses are strictly prohibited.).

### Bioinformatics Pipeline and Analysis

The software mothur 1.48.0 was used on the demultiplexed data to make contigs, using the *make.contig* function (Schloss et al., 2009). Prior to denoising, sequences within the 2nd and 95th percentiles in length (430 to 466 bp) were kept in the dataset, and sequences including homopolymers of 9 or more nucleotides were removed. Alignment was performed using SILVA and the script *align_seqs.py (**Caporaso* et al.*, 2010**).* Chimeric sequences were removed using the script *chimera.slayer* (mothur 1.48.0). Sequences were classified using the Ribosomal Database Project (RDP, mothur 1.48.0) trainset 14 (97 % similarity cut-off), and sequences corresponding with chloroplast, mitochondrial, unknown, archaeal and eukaryotic genes were filtered out of the dataset using the *remove.lineage* script (mothur 1.48.0). Sequences found within the blanks were removed from the samples data set. The remaining sequences were then assigned to Operational taxonomic units (OTUs) using the default settings to determine the quality of the clustering (*cluster.split,* OptiClust, mothur 1.48.0). Singletons were excluded from the dataset.

**Figure 1:**
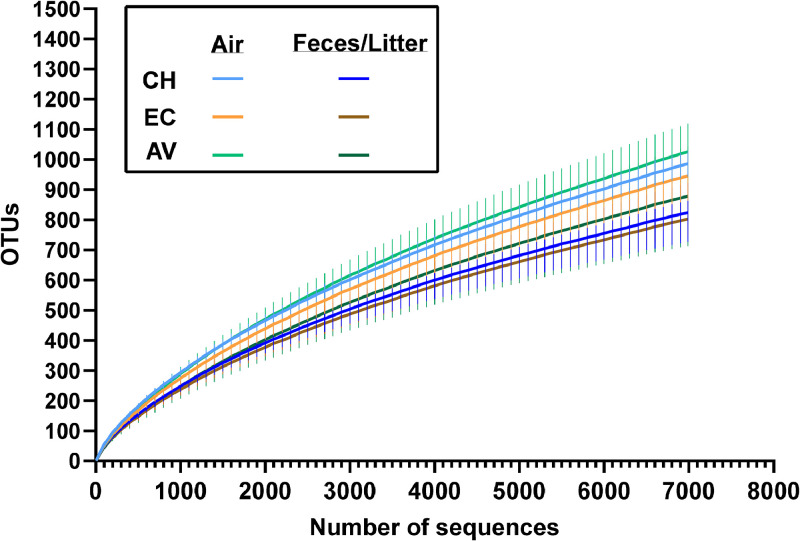
Rarefaction curves of air and droppings/litter samples from conventional hen house (CH), enriched colonies (EC) and aviaries (AV), mean ± SD.

Rarefaction curves were plotted using the *rarefaction.single* command in mothur. The final data sets equilibrated using the smallest number of reads (6,989 reads) (Figure 1).

The specific richness (S_obs_), the inverse Simpson index (InvSimpson), CHAO1 and Shannon indexes were used to describe alpha diversity, the diversity within each sample. The S_obs_ indicates the numbers of operational taxonomic units (OTUs) per sample. The inverse Simpson index (InvSimpson, 1/D) is based on the Simpson index (D), the latter which indicates the probability of sampling two microbes belonging to the same OTUs (Hugerth and Andersson, 2017; Simpson, 1949). A Simpson value of 0 would indicate an infinite diversity, while a value of 1 would indicate no diversity. In turn, a higher value of the inverse Simpson index indicates a higher diversity. InvSimpson also increases with OTUs richness and evenness among the samples. CHAO1 is a richness estimator using the specific richness (S_obs_) and considering species found only once (singleton) and twice (doubletons), being thus adapted for data sets with species with very low abundance (Chao, 1984; Hugerth and Andersson, 2017; Hughes et al., 2001). The CHAO1 calculator in mothur 1.48.0 allows for OTUs definition instead of specie. The Shannon index is the last diversity index used in this project. It describes how diverse the species are in the community, and increases with evenness (Hugerth and Andersson, 2017; Shannon, 1948).

### Statistical analyses

Mann-Whitney tests were performed to assess the differences in alpha diversity between the types of hen house for each sample type, and between sample types for each type of hen house. Multiple T-tests and analysis of molecular variance (AMOVA) were performed to assess the variance between the different sample groups. Bray-Curtis dissimilarity indices were used on the 50 most abundant OTUs within the dataset to assess differences between sample groups, and homogeneity of molecular variance (HOMOVA) tests were used to assess the homogeneity of variance among those groups. The *metastat* command in mothur with default settings was used to detect OTUs of different abundance among the air samples, for the 50 most abundance genus(White et al., 2009). P-values of < 0.05 were considered significant.

## Results and discussion

### Taxonomic attribution, coverage and alpha diversity indexes

Thirty-six field samples (air, feces or litter) and 36 blanks were analyzed. Rarefaction resulted in 6,989 sequences per sample. Samples had between 904 and 1,835 taxonomic attribution (Mean: 1,200; Standard Deviation (SD): 166) for a total of 43,202 taxonomic attributions within the dataset.

Coverage (one minus the number of OTUs sampled once, divided by the total number of individuals in the sample) was above 90 % in all samples for a maximum of 95. 29 % (Table 2). No significant differences were found between housing systems for both air, fecal or litter samples (*p* > 0.05). However, significant differences in coverage were found between air and fecal samples in conventional houses and enriched colonies (*p* < 0.05).

**Table 2:** Coverage (%) in air, fecal and litter samples of conventional hen houses, enriched colonies and aviaries (Mean ± SD, min-max) (*= *p* < 0.05, Mann-Whitney) (*n* = 36, 12 per housing system).

	Air	Feces/Litter	Air vs Feces/Litter P-value
**Conventional houses**	92.25 ± 0.35 (91.69 −92.79)	93.59 ± 1.09 (92.32–94.95)	*P* = 0.02 *
**Enriched colonies**	92.36 ± 0.80 (91.36-93.40)	93.65± 0.40 (93.12-94.09)	*P* = 0.01 *
**Aviaries**	91.76 ± 0.90 (90.21-92.76)	93.00 ± 1.58 (90.90-95.29)	*P* = 0.18

**Figure 2:**
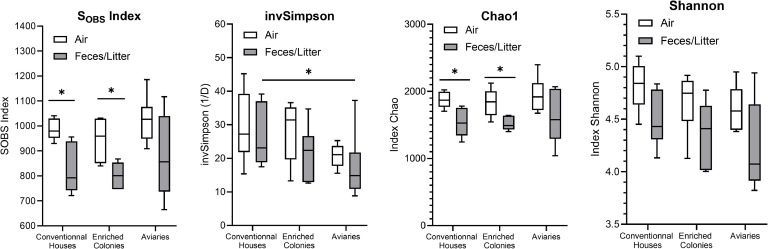
Alpha diversity indexes of air, fecal and litter samples from conventional hen houses, enriched colonies and aviaries. (mean, max-min) (* = *p* < 0.05, no mark = non-significant, Mann-Whitney).

S_obs_ index or Specific richness (amount of OTUs in a sample), Inverted Simpson index (InvSimpson), CHAO1 index and Shannon index for each sample group are shown in Figure 2. Higher S_obs_ indexes were found in air samples from conventional hen houses (986 ± 42, mean ±SD) and enriched colonies (946 ± 83, mean ±SD), compared to their corresponding fecal/litter samples (Conventional hen houses: 824 ± 98, mean ±SD; Enriched colonies: 802 ± 56, mean ±SD). No significant difference was found between air (1025 ± 93, mean ±SD) and litter (878 ± 165, mean ±SD) samples from aviaries. The InvSimpson indexes for the air and fecal/litter samples of their corresponding house type were not significantly different from one another. However, a significant difference was revealed between the feces from conventional houses (26 ± 6, mean ±SD) and the litter samples from aviaries (17 ± 10, mean ±SD). Akin to the S_obs_ indexes, the CHAO1 indexes for the air samples from conventional hen houses (1872 ± 119, mean ±SD) and enriched colonies (1833 ± 215, mean ±SD) were significantly higher than their corresponding fecal/litter samples (Conventional hen houses: 1533 ± 208, mean ±SD; Enriched colonies: 1517 ± 100, mean ±SD). By contrast, housing type had no significant effect on the S_obs_, InvSimpson, CHAO1 and Shannon indexes for both air and fecal/litter samples (*p* > 0.05, Figure S1 to S4, Supplementary Material). All sample groups had similar Shannon index (4.23 to 4.82).

Alpha diversity analyses show more richness (S_obs_ index) in air samples from conventional hen houses and enriched colonies than in their corresponding fecal samples. These results reflect the number of OTUs in the rarefied samples (Figure1) and could support the hypothesis that many sources contribute to the bioaerosol composition of hen houses (e.g., feces, skin, feathers, feed), which may result in more diversity found in the air than in a unique source. However, sampling of those potential sources would be needed to test this hypothesis. The InvSimpson, CHAO1 and Shannon indexes tend to show a similar trend of higher diversity in air samples, compared to their corresponding fecal/litter samples, but they are at odds when it comes to the statistical significance of the comparisons. These differences may be attributed to the assumptions underlying each index. The Simpson index is less affected by OTUs of lesser abundance (Simpson, 1949), while the Shannon index assumes all taxa are represented in a sample and that individuals are randomly sampled (Hugerth and Andersson, 2017; Shannon, 1948). CHAO1 may more adequately represent alpha diversity herein, as it assumes a Poisson distribution, which may be more representative of the dataset (with very abundant OTUs and OTUs with very low abundance) (Chao, 1984; Chao and Bunge, 2002; Hughes et al., 2001). It can also be noted that the comparisons between the Shannon indexes of air and fecal/litter samples were close to statistical significance (α = 0.05) (Fig S4), following the tendency of the CHAO1 index. A larger number of samples could have strengthened statistical power enough to reach statistical significance.

### OTUs distribution and beta diversity

**Figure 3:**
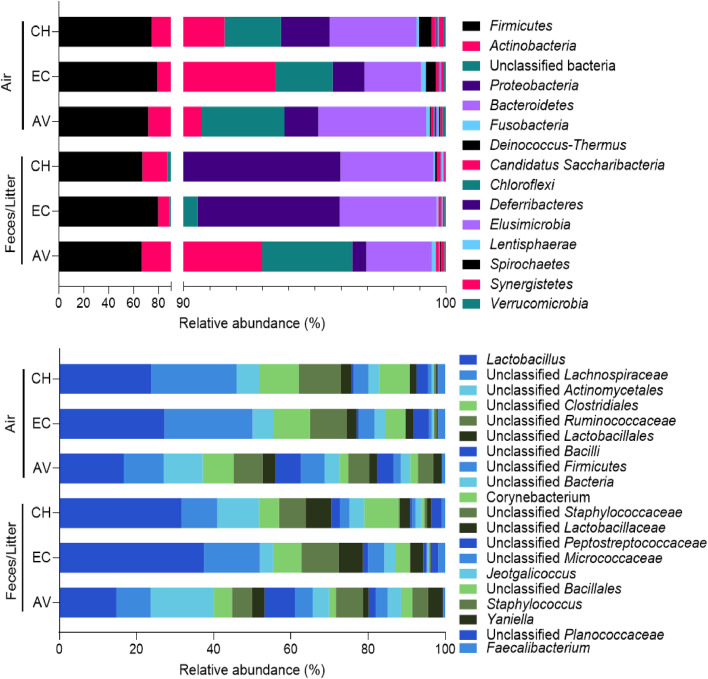
Relative abundance of the 15 most abundant phyla (top) and the 20 most abundant genera (bottom) in air and fecal/litter samples from conventional housing systems (CH), enriched colonies (EC) and aviaries (AV).

Across all sample groups, *Firmicutes* (*Bacillota*) were the most abundant phylum, with a mean relative abundance ranging from 66.22 % to 79.51 % of the 15 most abundant phyla (Figure 3). Actinobacteria was the second most abundant phylum, with mean relative abundance between 8.86 % and 26.77 %. Other phyla representing more than 1 % relative abundance are the unclassified bacteria, *Proteobacteria* and *Bacteroidetes* (*Bacteriodota*). Phyla representing less than 1 % relative abundance were fusobacteria, *Deinococcus/Thermus, Candidatus - Saccharibacteria, Chloroflexi, Deferribacteres, Elusimicrobia, Lentisphaerae, Spirochaetes, Synergistetes* and *Verrucomicrobia*.

The genus *Lactobacillus* had a relative abundance of 14.73 % to 37.39 % across all sample groups. The second and third most abundant genera were unclassified (uncl.) *Lachnospiraceae* (8.92 % to 22.81 % relative abundance) and uncl. *Actinomycetales* (3.43 % to 16.43 % relative abundance). The other most abundant genera and unclassified OTUs at a higher taxonomic level were uncl. *Clostridiales,* uncl. *Ruminococcaceae,* unl. *Lactobacillales,* uncl. *Bacilli,* uncl. *Firmicutes,* uncl. *Bacteria, Corynebacterium,* uncl. *Staphylococcaceae,* uncl. *Lactobacillaceae,* uncl. *Peptostreptococcaceae,* uncl. *Micrococcaceae, Jeotgalicoccus,* uncl. *Bacillales, Staphylococcus, Yaniella,* uncl. *Planococcaceae* and *Faecalibacterium.*

**Figure 4:**
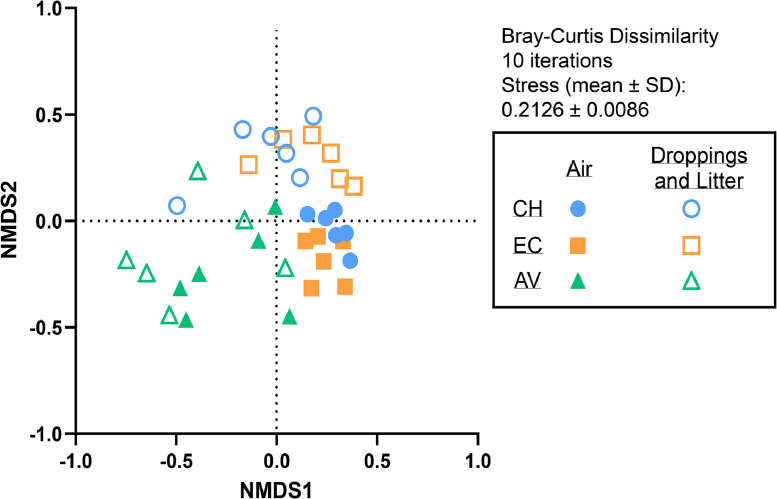
Non-metric multidimensional scaling (NMDS) ordination of air and fecal/litter samples from conventional hen houses (CH), enriched colonies (EC) and aviaries (AV), from Bray-Curtis indices of top 50 OTUs.

The HOMOVA analysis revealed significant differences between the air samples of aviaries, compared to those from conventional hen houses (*p* < 0.001) and enriched colonies (*p* = 0.01). Significant differences were also found between air samples and the corresponding fecal samples in conventional hen houses (*p* = 0.003) and enriched colonies (*p* = 0.035). However, no significant differences were found between air samples from aviaries and their corresponding litter samples (*p* = 0.245), nor were there any between fecal/litter samples from each of the studied housing types (CH vs CE: *p* = 0.933, CH vs AV: *p* = 0.057, EC vs AV: *p* = 0.096) (Figure S6. supplementary material). Sample groups were plotted on a NMDS using the Bray-Curtis indices (Figure 4), showing air samples (filled shapes) and feces/litter samples (empty shapes) from conventional hen house (blue circles), enriched colonies (orange squares) and aviaries (green triangle). The distance between those sample groups show the dissimilarity between each other (ie. Air samples from aviaries vs air samples from conventional hen house and enriched colonies). While overlapping groups show more similarity with one another (ie. Air dans feces samples in conventional hen houses).

The OTUs contributing to the differences between the air sample groups are detailed in Table S1 and Table S2 (Supplementary material). *Staphylococcus, Salinicoccus Brevibacterium,* unclassified *Dermabacteraceae* and *Escherichia/Shigella* were more abundant in bioaerosols from aviaries than in conventional hen houses bioaerosols. Whereas the OTUs *Lactobacillus,* unclassified *Lachnospiraceae, Faecalibacterium, Blautia,* unclassified *Ruminococcaceae, Corynebacterium, Aeriscardovia, Rothia, Kocuria, Facklamia, Aerococcus, Trichococcus,* and *Gallicola* were more abundant in the air of conventional hen houses than in aviaries. The genera *Staphylococcus, Salinicoccus, Brevibacterium* and unclassified *Dermabacteraceae* were more abundant in the bioaerosols from aviaries than in those from enriched colonies. The OTUs most abundant in enriched colonies were *Lactobacillus,* unclassified *Lachnospiraceae, Faecalibacterium, Blautia, Aerococcus,* unclassified *Ruminococcaceae* and *Gallicola.*

Genera, families and other taxonomic rank attributions were associated with chicken gut and skin microbiota, and soil. The dominance of *Firmicutes* and the presence of *Actinobacteria, Fusobacteria* and *Bacteriodetes* were previously reported in conventional laying hen bioaerosols (Cui et al., 2023) and cecal microbiota (Bari et al., 2022; Khan et al., 2020; Rychlik, 2020; Wang et al., 2023). *Lactobacillus,* unclassified *Lachnospiraceae,* unclassified *Clostridiales, Ruminococcaceae,* unclassified *Lactobacillales,* unclassified *Bacilli,* unclassified *Peptostreptococcaceae, Staphylococcus and Faecalibacterium* were reported in chicken or hen gut microbiota (Bari et al., 2022; Khan et al., 2020; Rychlik, 2020; Wang et al., 2023), while *Jeotgalicoccus* was reported in laying hen house bioaerosols (Cui et al., 2023). Despite these differences, most OTUs were shared by all air samples (Table S1 and Table S2, Supplementary material), and associated with either chicken gut or skin microbiota (e.g., *Lactobacillus, Staphylococcus, Fusobacterium, Lachnospiraceae, Blautia, Aesriscardiovia, Rothia* and *Gallicola)* (Bindari et al., 2021; Eeckhaut et al., 2008; Ezaki et al., 2001; Farooq et al., 2023; Garrity et al., 2006; Khan et al., 2020; Krieg et al., 2011; Vos et al., 2009; Whitman et al., 2012; Zhang et al., 2022). The same was observed in the twenty most abundant genera in air and fecal samples, albeit in different proportions. Thus, observed differences between hen house bioaerosols did not coincide with the presence or absence of specific genera, but to their proportion in the samples (Table S1 and Table S2, Supplementary material). Aviary bioaerosols and litter samples showed greater similarity to one another, as illustrated in the NMDS ordination in Figure 4. This, and the dominant genera found in both sample groups, could indicate a possible contribution of litter to bacterial diversity in the air, as hens regularly interact with the litter surface by foraging, walking, dustbathing and taking flight (Arteaga et al., 2015; Le Bouquin et al., 2013; National Farm Animal Care Council, and Egg Farmers of Canada, 2017), leading to the aerosolization of its biological content. In contrast, hen housed in conventional hen house or in enriched colonies are physically separated from their feces by the mesh enclosures, which are designed to let feces fall onto manure belts. This could lead to less aerosolization of the feces’ content in the hen house, and have an impact on airborne bacterial diversity. Those shared features between conventional and enriched hen houses may explain their similar bacterial diversity and the lesser overlap between air and feces samples in those environments.

High-flow air sampling allowed for the retrieval of bioaerosols in conventional and alternative hen houses in Eastern Canada. High-flow sampling on electret filter was effective in retrieving air particulates from animal husbandry for molecular analysis (Bergeron et al., 2024; Mbareche et al., 2018, 2019a, 2019b; Pilote et al., 2019). Triplicates from the same sampling campaign were then pooled (equimolar) to conduct high-throughput sequencing, so to represent the 4-hour air sampling period. The number of barns visited for each housing type led to enough data points to conduct appropriate statistical analyses.

Samplings were conducted during the months of October up to April, in flocks with 55 to 65 weeks of age and in a single location within each house, away from air inlets and outlets to limit the influence of outdoor air and air drafts on the samples. The sampling months were selected for a previous study investigating airborne dusts and bioaerosols concentrations in the same hen houses (St-Germain et al., 2024b), as lower ventilation rates were expected, so to preserve indoor temperature within the hen houses during colder seasons (median outdoor temperatures between −6.60°C to −0.550, median indoor temperatures between 21.4°C and 22.6°C). Likewise, indoor relative humidity (RH) and ventilation rates were similar between laying hen house types (median; conventional hen house: 57.2 % RH, 0.660 m³/h/hen; enriched colonies: 54.9 % RH, 0.595 m³/h/hen; aviaries: 57.3 % RH, 0.840 m³/h/hen; *p* > 0.05, Mann-Whitney, GraphPad Prism 10.4.2). Sampling months, flock age and sampling location were applied similarly to all sampled hen house, thus allowed for comparisons between housing types in those specific conditions.

Though the effect of the season of hen age on airborne bacterial diversity in 24 other enriched colonies (*n* = 12) and aviaries (*n* = 12) in Eastern Canada (St-Germain et al., 2024a), further studies would be needed to thoroughly investigate other factors and sources (ex. feathers, skin, feed) which may impact airborne bacterial diversity in alternative hen houses. Sampling at multiple locations within hen houses would allow the assessment of spatial variations within buildings, as investigated in caged-broiler houses (Yan et al., 2023). Composite samples of feces and litter, taken at randomized locations, may have given a more representative portrait of bacterial diversity in hen house feces and litter as well.

## Conclusions

High-throughput sequencing was used to study bacterial diversity in the bioaerosols and feces/litter from six conventional hen houses (battery cages), six enriched colonies (enriched cages) and six aviaries (cage-free housing with multi-tier perches). The effects of housing and sample type were assessed in the present study. Air samples from all housing types generally had higher alpha diversity compared to their corresponding fecal/litter samples. Bacterial diversity was significantly different between air and fecal/litter samples from conventional housing and enriched colonies. However, no significant difference was observed between the bioaerosols and the fecal/litter samples from aviaries. Bioaerosol diversity composition was similar in conventional hen houses and in enriched colonies, while significant differences were found between aviaries and cage houses (conventional and enriched). Differences found between sample groups coincided to the OTUs’ relative abundance, and rather than the presence or absence of specific OTUs in any given group.

Further studies are needed to investigate the impact of additional factors relative to housing, management practices and other sources on airborne bacterial diversity, the presence of specific pathogenic agents or avian viruses, and their effect on flock health.

## Declaration of competing interest

The authors declare no conflict of interests.

## Appendix Supplementary materials

Image, application 1
