# Supplementary material for: Characterization of airborne bacterial diversity in conventional hen houses, enriched colonies and aviaries, and link between possible bioaerosol sources
Source: Poult Sci. 2025 Apr 29;104(8):105217. doi: 10.1016/j.psj.2025.105217 (PMC12144446; doi:10.1016/j.psj.2025.105217)
Supplement: Supplementary file 1 [file mmc1.docx]

Characterization of airborne bacterial diversity in conventional hen houses, enriched colonies and aviaries , and link between possible bioaerosol sources

M.-W. St-Germain^*^, M. Veillette^#^, V. Létourneau^#^, A.D. Larios Martínez^##^, S. Godbout^##^, M. Boulianne^§###^, C. Duchaine ^*#||^

** Département de biochimie, de microbiologie et de bio-informatique, Faculté des sciences et de génie, Université Laval, Québec, Canada*

*# Centre de recherche de l’Institut universitaire de cardiologie et de pneumologie de Québec, Québec, Canada*

*## Research and Development Institute for the Agri-Environment (IRDA), Québec, Canada*

*§ Department of Clinical Sciences, Faculty of Veterinary Medicine, Université de Montréal, Saint-Hyacinthe, Canada*

*### Chaire en recherche avicole de l'Université de Montréal, Faculty of Veterinary Medicine, Université de Montréal, Saint-Hyacinthe, Canada*

^||^ *Canada Research Chair on Bioaerosols, Québec, Canada*

**For the journal**

**Poultry Science**

Corresponding author: [caroline.duchaine@ulaval.ca](mailto:caroline.duchaine@ulaval.ca)

|  | | **Air** | | | **Feces/Litter** | | |
| --- | --- | --- | --- | --- | --- | --- | --- |
|  |  | CH | EC | AV | CH | EC | AV |
| **Air** | CH |  | >0.99 | 0.37 | 0.02 |  |  |
|  | EC |  |  | 0.39 |  | 0.01 |  |
|  | AV |  |  |  |  |  | 0.18 |
| **Feces/Litter** | CH |  |  |  |  | 0.68 | 0.46 |
|  | EC |  |  |  |  |  | 0.29 |
|  | AV |  |  |  |  |  |  |

**Figure S1. P-values of Mann-Whitney analyses for coverage values (Yellow = P< 0.05). CH: Conventional housing systems; EC: Enriched colony housing; AV: Aviaries**

|  | | **Air** | | | **Feces/Litter** | | |
| --- | --- | --- | --- | --- | --- | --- | --- |
|  |  | CH | EC | AV | CH | EC | AV |
| **Air** | CH |  | 0.59 | 0.59 | 0.01 |  |  |
|  | EC |  |  | 0.18 |  | 0.03 |  |
|  | AV |  |  |  |  |  | 0.09 |
| **Feces/Litter** | CH |  |  |  |  | 0.78 | 0.59 |
|  | EC |  |  |  |  |  | 0.41 |
|  | AV |  |  |  |  |  |  |

**Figure S2. P-values of Mann-Whitney analyses for SOBS index values (Yellow = P< 0.05). CH: Conventional housing systems; EC: Enriched colony housing; AV: Aviaries**

|  | | **Air** | | | **Feces/Litter** | | |
| --- | --- | --- | --- | --- | --- | --- | --- |
|  |  | CH | EC | AV | CH | EC | AV |
| **Air** | CH |  | 0.82 | 0.13 | 0.70 |  |  |
|  | EC |  |  | 0.18 |  | 0.24 |  |
|  | AV |  |  |  |  |  | 0.09 |
| **Feces/Litter** | CH |  |  |  |  | 0.39 | 0.04 |
|  | EC |  |  |  |  |  | 0.39 |
|  | AV |  |  |  |  |  |  |

**Figure S3. P-values of Mann-Whitney analyses for invSimpson index values (Yellow = P< 0.05) CH: Conventional housing systems, EC: Enriched colony housing, AV: Aviaries**

|  | | **Air** | | | **Feces/Litter** | | |
| --- | --- | --- | --- | --- | --- | --- | --- |
|  |  | CH | EC | AV | CH | EC | AV |
| **Air** | CH |  | 0.70 | 0.70 | 0.01 |  |  |
|  | EC |  |  | 0.82 |  | 0.01 |  |
|  | AV |  |  |  |  |  | 0.18 |
| **Feces/Litter** | CH |  |  |  |  | 0.94 | 0.59 |
|  | EC |  |  |  |  |  | 0.82 |
|  | AV |  |  |  |  |  |  |

**Figure S4. P-values of Mann-Whitney analyses for Chao1 index values (Yellow = P< 0.05) CH: Conventional housing systems, EC: Enriched colony housing, AV: Aviaries**

|  | | **Air** | | | **Feces/Litter** | | |
| --- | --- | --- | --- | --- | --- | --- | --- |
|  |  | CH | EC | AV | CH | EC | AV |
| **Air** | CH |  | 0.31 | 0.13 | 0.06 |  |  |
|  | EC |  |  | 0.48 |  | 0.06 |  |
|  | AV |  |  |  |  |  | 0.09 |
| **Feces/Litter** | CH |  |  |  |  | 0.59 | 0.31 |
|  | EC |  |  |  |  |  | 0.39 |
|  | AV |  |  |  |  |  |  |

**Figure S5. P-values of Mann-Whitney analyses for Shannon index values (Yellow = P< 0.05). CH: Conventional housing systems; EC: Enriched colony housing; AV: Aviaries**

|  | | **Air** | | | **Feces/Litter** | | |
| --- | --- | --- | --- | --- | --- | --- | --- |
|  |  | CH | EC | AV | CH | EC | AV |
| **Air** | CH |  | 0.059 | <0.001* | 0.003* | 0.003 | 0.004 |
|  | EC |  |  | 0.012 | 0.09 | 0.035* | 0.012 |
|  | AV |  |  |  | 0.251 | 0.293 | 0.245 |
| **Feces/Litter** | CH |  |  |  |  | 0.933 | 0.057 |
|  | EC |  |  |  |  |  | 0.096 |
|  | AV |  |  |  |  |  |  |

**Figure S6. P-values of HOMOVA analyses for the comparison of the sample groups, using the 50 most abundant OTUs (Yellow = P< 0.05). CH: Conventional housing systems; EC: Enriched colony housing; AV: Aviaries**

| **Table S1. Affiliation of the OTUs contributing to the differences between air samples of aviaries and air samples of conventional hen houses among the top 50 most abundant genera** | | | |
| --- | --- | --- | --- |
| **OTUs**  **Genus (identity %) – Phyla (identity%)** | **Average % in aviaries** | **Average % in conventional hen houses** | **p-value** |
| *Staphylococcus* (100) *-Firmicutes (100)* | **11.79%** | 0.12% | 0.002 |
| *Salinicoccus* (90) *-Firmicutes (100)* | **6.78%** | 0.00% | 0.036 |
| *Brevibacterium* (89)-*Actinobacteria (100)* | **1.40%** | 0.03% | 0.019 |
| Unclassified *Dermabacteraceae* (63)-*Actinobacteria (100)* | **0.80%** | 0.00% | 0.032 |
| *Escherichia/Shigella* (100)-*Proteobacteria* | **0.04%** | 0.01% | 0.021 |
| *Lactobacillus* (100)-*Firmicutes (100)* | 6.58% | **19.96%** | < 0.001 |
| Unclassified *Lachnospiraceae* (69)*-Firmicutes (100)* | 2.58% | **10.28%** | < 0.001 |
| *Faecalibacterium* (100)*-Firmicutes (100)* | 1.23% | **4.76%** | 0.002 |
| *Lactobacillus* (100)*-Firmicutes (100)* | 0.99% | **3.05%** | 0.002 |
| Unclassified *Lachnospiraceae* (100) *-Firmicutes (100)* | 0.72% | **2.03%** | 0.018 |
| Unclassified *Lachnospiraceae* (95)*-Firmicutes (100)* | 0.70% | **2.40%** | 0.002 |
| *Blautia* (93)*-Firmicutes (100)* | 0.60% | **1.43%** | 0.008 |
| Unclassified *Ruminococcaceae* (99)*-Firmicutes (100)* | 0.50% | **0.95%** | 0.033 |
| Unclassified *Lachnospiraceae* (100)*-Firmicutes (100)* | 0.47% | **1.19%** | 0.001 |
| Unclassified *Ruminococcaceae* (80)*-Firmicutes (100)* | 0.42% | **1.34%** | 0.001 |
| *Corynebacterium* (100)*-Actinobacteria (100)* | 0.37% | **2.47%** | 0.010 |
| *Lactobacillus* (100)*-Firmicutes (100)* | 0.34% | **1.08%** | 0.002 |
| Unclassified *Ruminococcaceae* (74)*-Firmicutes (100)* | 0.20% | **1.04%** | 0.000 |
| *Corynebacterium* (100)*-Actinobacteria (100)* | 0.18% | **2.60%** | 0.013 |
| *Aeriscardovia* (100)*-Actinobacteria (100)* | 0.10% | **0.97%** | 0.003 |
| *Rothia* (92)*-Actinobacteria (100)* | 0.09% | **2.14%** | 0.021 |
| *Kocuria* (90)*-Actinobacteria (100)* | 0.08% | **2.04%** | 0.009 |
| *Facklamia* (100)*-Firmicutes (100)* | 0.08% | **0.46%** | 0.008 |
| *Aerococcus* (100)*-Firmicutes (100)* | 0.06% | **0.65%** | 0.008 |
| *Trichococcus* (69)*-Firmicutes (100)* | 0.02% | **0.22%** | 0.003 |
| *Gallicola* (100)*-Firmicutes (100)* | 0.02% | **0.66%** | 0.001 |

| **Table S2. Affiliation of the OTUs contributing to the differences between air samples of aviaries and air samples of enriched colonies among the top 50 most abundant genera** | | | |
| --- | --- | --- | --- |
| **OTUs**  **Genus (identity %) – Phyla (identity%)** | **Average % in aviaries** | **Average % in enriched colonies** | **p-value** |
| *Staphylococcus* (100)*-Firmicutes (100)* | **11.79%** | 0.69% | 0.002 |
| *Salinicoccus* (90)*-Firmicutes (100)* | **6.78%** | 0.00% | 0.041 |
| *Brevibacterium* (89)*- Actinobacteria (100)* | **1.40%** | 0.05% | 0.025 |
| Unclassified *Dermabacteraceae* (63)*-Actinobacteria (100)* | **0.80%** | 0.00% | 0.037 |
| *Lactobacillus* (100)*-Firmicutes* (100) | 6.58% | **16.37%** | 0.004 |
| Unclassified *Lachnospiraceae* (69)*-Firmicutes* (100) | 2.58% | **7.89%** | 0.001 |
| *Faecalibacterium* (100)*-Firmicutes* (100) | 1.23% | **6.29%** | 0.007 |
| *Lactobacillus* (100)*-Firmicutes* (100) | 0.99% | **4.96%** | 0.007 |
| Unclassified *Lachnospiraceae* (95)*-Firmicutes* (100) | 0.70% | **2.69%** | < 0.001 |
| *Blautia* (93)*-Firmicutes (100)* | 0.60% | **2.04%** | < 0.001 |
| *Aerococcus* (100)*-Firmicutes* (100) | 0.06% | **1.70%** | 0.005 |
| Unclassified *Ruminococcaceae* (80)*-Firmicutes* (100) | 0.42% | **1.68%** | 0.001 |
| Unclassified *Lachnospiraceae* (100)*-Firmicutes* (100) | 0.47% | **1.23%** | 0.001 |
| Unclassified *Lachnospiraceae* (98)*-Firmicutes* (100) | 0.22% | **1.07%** | 0.011 |
| *Lactobacillus* (100)*-Firmicutes* (100) | 0.34% | **0.93%** | 0.010 |
| Unclassified *Ruminococcaceae* (74)*-Firmicutes* (100) | 0.20% | **0.93%** | 0.004 |
| *Gallicola* (100)*-Firmicutes* (100) | 0.02% | **0.15%** | 0.024 |
